# Supplementary material for: Gastroprotective Effect of Isoferulic Acid Derived from Foxtail Millet Bran against Ethanol-Induced Gastric Mucosal Injury by Enhancing GALNT2 Enzyme Activity
Source: Nutrients. 2024 Jul 5;16(13):2148. doi: 10.3390/nu16132148 (PMC11243359; doi:10.3390/nu16132148)
Supplement: Supplementary file 1 [file nutrients-16-02148-s001.zip › nutrients-3072999-supplementary.pdf]

Table S1. The Compounds of BPIS.

No.	RT [min]	Class of compound	Name	Molecular formula	[M-H]-	MW
1	7.48	Polyphenols	4-Hydroxybenzoic acid	$C_7H_6O_3$	137	138
2	10.8	Polyphenols	*p*-Coumaric acide	$C_9H_8O_3$	165	166
3	8.51	Polyphenols	Vanillic acid	$C_8H_8O_4$	167	168
4	12.3	Polyphenols	Isoferulic acid	$C_{10}H_{10}O_4$	193	194
5	11.7	Polyphenols	Ferulic acid	$C_{10}H_{10}O_4$	193	194
6	8.97	Polyphenols	Syringic acid	$C_9H_{10}O_5$	197	198
7	4.75	Polyphenols	Vanillic acid 4-*O*-β-D-glucopyranoside	$C_{14}H_{18}O_9$	329	330
8	7.0	Polyphenols	Ferulic acid 4-*O*-β-D-glucopyranoside	$C_{16}H_{20}O_9$	355	356
9	5.56	Polyphenols	Glucosyringic acid	$C_{15}H_{20}O_{10}$	359	360
10	16.4	Polyphenols	4,4′-Dihydroxy-3,5′-dimeth oxy,3′-bicinnamc acid	$C_{20}H_{18}O_8$	385	386
11	14.2	Polyphenols	Biferulic acid	$C_{20}H_{18}O_9$	401	402
12	11.3	Polyphenols	Vitexin	$C_{21}H_{20}O_{10}$	431	432

Table S2. The scoring criteria of strip injury.

Scoring criteria of strip injury		Scoring criteria of flaky injury	
Length of damaged part	Scoring	Damage area	Scoring
Normal mucosa	0	1-12 mm^2	1
Every spot bleeding	1	13-25 mm^2	2
The length of each damage is less than 1 mm	2	26-37 mm^2	3
The length of each damage is between 1 mm and 2 mm	3	38-50 mm^2	4
The length of each damage is between 2 mm and 4 mm	4	Greater than 51 mm^2 or perforated	5
The length of each damage is greater than 4 mm	5	Ulcer area=$\pi \times$ (Length $\times$ Width)/4	
Damage width greater than 2 mm	Multiply the score by 2		

Table S3. The scoring criteriaof gastric mucosal pathological damage.

The damage of gastric mucosa	Scoring
Gastric superficial epithelial cell injury	1
edema and congestionin the upper gastric mucosa	2
edema and congestion in the middle and lower of gastric	3
Necrosis and disorder of the upper gland structure of the	4
Deep ulcer and necrosis of gastric mucosa	5

Table S4. Sequence of Forward and Rerverse primers in

MUC5AC、MUC6、GAPDH.

Primer name		primer sequences
MUC5AC	Forward	5′-TCACTCTACCACTCCCTGCTTCTG-3′
	Reverse	5′-CACCTGACAATCCTGGCTACACATC-3′
MUC6	Forward	5′-CATCCTACACGACGCAGCATCAG-3′
	Reverse	5′-TGGCAACGAGTTAGAGTCACATTGG-3′
GAPDH	Forward	5′-GCACCGTCAAGGCTGAGAAC-3′
	Reverse	5′-TGGTGAAGACGCCAGTGGA-3′
